# *Klebsiella pneumoniae* bloodstream infections at a South African children’s hospital 2006–2011, a cross-sectional study

**DOI:** 10.1186/s12879-016-1919-y

**Published:** 2016-10-17

**Authors:** Heloise Buys, Rudzani Muloiwa, Colleen Bamford, Brian Eley

**Affiliations:** 1 Department of Paediatrics and Child Health, Red Cross War Memorial Children’s Hospital, Klipfontein Road, Cape Town, Rondebosch 7700 South Africa; 2 University of Cape Town, Cape Town, South Africa; 3 Ambulatory and Emergency Paediatrics, Red Cross War Memorial Children’s Hospital, Klipfontein Road, Cape Town, Rondebosch 7700 South Africa; 4 National Health Laboratory Service (Groote Schuur Hospital), Cape Town, South Africa

**Keywords:** *Klebsiella pneumoniae* bloodstream infection, Children, Africa

## Abstract

**Background:**

*Klebsiella pneumoniae* (KP) is a significant paediatric bloodstream pathogen in children. There is little data from Africa. In this study we describe the epidemiology of multi-drug resistant *Klebsiella pneumoniae* bloodstream infection (KPBSI) at Red Cross War Memorial Children’s Hospital, Cape Town, South Africa.

**Methods:**

We conducted a retrospective cross-sectional study of KPBSI from 1 January 2006 to 31 December 2011 using conventional descriptive and inferential statistical methods.

**Results:**

Of 410 hospitalised children with laboratory confirmed KPBSI, 339 (83 %) were caused by extended-spectrum β-lactamase (ESBL) producing isolates. The median age (IQR) was 5.0 (2–16) months, 212 (51.7 %) were male, 82 (20 %) were HIV-infected, and 241 (58.8 %) were moderately or severely underweight. The infection was hospital-acquired or healthcare-associated in 389 (95 %) children and community-acquired in 21 (5 %) children. Significant risk factors for ESBL-KPBSI included cephalosporin exposure in the 12 months prior to the KPBSI, adjusted risk ratio (aRR) 1.18 (95 % CI: 1.06–1.31); HIV infection, aRR 1.14 (1.04–1.25), and intravenous infusions for more than 3 days before the KPBSI, aRR 1.15 (95 % CI: 1.04–1.28).

A total of 109 (26.6 %) children died within 30 days of the KPBSI, their median age was four (IQR 1–11) months. The median (IQR) time between KPBSI and death was three (1–9) days. HIV-infection, aRR 2.44(95 % CI: 1.59–3.74); skin erosions at the time of KPBSI, aRR 2.15 (95 % CI: 1.54–3.00); being in PICU at the time of the KPBSI, aRR 1.64 (95 % CI: 1.03–2.61) or needing PICU admission after developing KPBSI, aRR 1.72 (95 % CI: 1.10–2.70) were significant risk factors for death.

**Conclusion:**

ESBL-producing KP is an important cause of laboratory confirmed bloodstream infection in hospitalised children and is associated with high mortality.

## Background

*Klebsiella pneumoniae* (KP) is a significant bloodstream pathogen resulting in major treatment and cost challenges for health institutions [1]. There is little data on children from Africa where the effects of socioeconomic inequalities and inadequate healthcare delivery remain challenging and influence the outcome of infectious diseases.

International reports show that extended-spectrum β-lactamase (ESBL) producing Gram-negative bloodstream infections (BSI) occur mainly in the setting of hospital exposure. Reported risk factors for ESBL-strain acquisition in paediatric Gram-negative BSI including KP include prior cephalosporin exposure [2–6], prior hospitalisation [3, 5], prolonged hospitalisation [7], previous corticosteroid use [2, 6], prolonged total parenteral nutrition [8], paediatric intensive care unit (PICU) admission [3], presence of a central venous pressure (CVP) line [6], neutropaenia [6], and underlying renal disease [4].

The outcome data from the limited number of paediatric studies involving Gram-negative BSI including KP portray high mortality ranging from 12.9 to 36 % [2–5, 7–10].

Over the last few years anecdotal observation suggested that ESBL-producing KP frequently caused invasive infection at our hospital. We therefore reviewed laboratory-confirmed KPBSI from 1 January 2006 to 31 December 2011 to document the extent of the problem at our hospital. The specific objectives of the study were to describe the clinical presentation of KPBSI, risk factors associated with ESBL-KPBSI, antibiotic susceptibility patterns of the KP isolates and KPBSI mortality including factors associated with in-patient mortality.

## Methods

### Study design and setting

This hospital based retrospective case folder review was conducted at Red Cross War Memorial Children’s Hospital (RCWMCH), Cape Town, Western Cape province, South Africa. RCWMCH is a 282- bedded public children’s hospital for children aged 0–13 years; it is also an academic teaching hospital of the University of Cape Town and is one of two local tertiary referral units that service 1.5 million children <14 years of age in the Western Cape. An average of 3500 new children per month (range 3000 to 6000 per month) are evaluated in its medical emergency unit, and the total number of hospital admissions peaks at 23 000 children per annum. There is a 20-bedded multidisciplinary PICU, an 18-bed burn unit, 13-bed oncology unit, 60 mixed surgical beds, 58 general paediatric beds; 29-bed cardiology and tracheostomy ward, 29 specialty medical beds, 10-bed trauma unit and 42 short stay medical beds. Dedicated newborn care is available at other hospitals in Cape Town where obstetric care is provided. Admission numbers peak during diarrhoeal seasonal months (December to April) and overcrowding commonly occurs throughout the hospital. The main entry points into the hospital are via the medical emergency or trauma units.

### Study population

Most of the children admitted to RCWMCH come from homes in low socioeconomic, peri-urban settlements in the Western Cape Province. The Medical Microbiology laboratory of the National Health Laboratory Service (NHLS) identified and forwarded an electronic list of *Klebsiella pneumoniae* bloodstream isolates identified between 1 January 2006 and 31 December 2011 for children admitted to RCWMCH.

### Data collection

Data were collected from paper-based medical records including patient demographics, admission diagnosis, HIV and nutrition status, age, date of current hospital admission, underlying medical condition, previous medical history, antibiotic history, site of KPBSI, potential risk factors for ESBL-KPBSI and mortality, length of hospital stay and outcome.

### Laboratory procedures

All microbiology testing was conducted at the Groote Schuur National Health Laboratory Service (NHLS) microbiology laboratory. The laboratory used the BACTEC™ 9240 automated blood culture system (Becton Dickinson, Sparks, Maryland. For blood culture bottles flagging positive with mono-morphic Gram-negative bacilli seen on Gram stain, a locally validated method of direct inoculation into the automated Vitek®2 system (bioMérieux, Inc, France) was employed, [11] using Vitek® ID-GNB and AST-N133 cards for identification and susceptibility testing respectively. This direct method was supplemented, where necessary with repeat testing from bacterial colonies subcultured onto agar plates, using either Vitek, disk diffusion or E-test (bioMérieux, Marcy l’Etoile, France) methods. The following antibiotics were tested: ampicillin, co-amoxyclav, piperacillin-tazobactam, cefuroxime, cefoxitin, ceftriaxone, ceftazidime, cefepime, ertapenem, meropenem, imipenem, ciprofloxacin, gentamicin, amikacin, cotrimoxazole, tigecycline and colistin. Susceptibility results were interpreted according to the Clinical Laboratory Standards Institute (CLSI) criteria for the relevant years [12]. ESBLs were detected by the Vitek® 2 Advanced Expert system or by the double disk synergy method if using disk diffusion testing. Despite the changes to reporting of cephalosporin susceptibility in ESBL-producing organisms introduced by CLSI in 2010, the laboratory, in line with the contemporary national practice, continued to report ESBL- producing *Enterobacteriaceae* as resistant to all cephalosporins. Since ESBL production was not explicitly reported on the laboratory information system (LIS) due to limitations in the design and setup of the interface, ESBL production was assumed if *Klebsiella pneumoniae* isolates were reported to the clinicians (on the LIS) as resistant to all cephalosporins.

### Definitions

#### Hospital-acquired Klebsiella pneumoniae Blood Stream infection (HA-KPBSI)

*Klebsiella pneumoniae* blood stream infection detected 48 h or more after hospital admission and not incubating at the time of hospitalisation [13].

#### Healthcare-associated (HCA)-KPBSI

*Klebsiella pneumoniae* blood stream infection detected within 48 h of hospital admission in children who have had contact with the healthcare service including admission to an intermediate care facility during the 12-month period preceding hospitalisation. An intermediate facility provides step-down care for children recovering from acute illness, for a maximum duration of 6 weeks [13].

#### Community-acquired (CA)-KPBSI

*Klebsiella pneumoniae* blood stream infection detected within 48 h of hospital admission without previous contact with the healthcare service.

#### ESBL-producing KP (ESBL KP)

*Klebsiella pneumoniae* isolates were classified as ESBL-producing if they were resistant to cefepime [13].

#### HIV infection

A positive HIV DNA PCR result confirmed by either a HIV RNA PCR or repeat HIV DNA PCR test, in any child < 18 months old, or 2 positive serological test results (HIV ELISA or HIV Rapitest) or a positive HIV DNA PCR result confirmed by either a HIV RNA PCR or repeat HIV DNA PCR test, in a child > 18 months old were considered HIV-infected [14].

#### Unknown HIV status

Any infant of child where there was no record of HIV testing at the NHLS laboratory database and whose mother’s HIV status was unknown.

#### Severe immunosuppression

A CD4 percentage and / or an absolute CD4 count below the age defined range in an HIV-infected child, using the 2006 WHO definitions [15].

#### Nutritional status

Moderate and severe underweight were defined as weight-for-age z score (WAZ) between −2 and −3 standard deviations (SD) below the median World Health Organisation (WHO) growth reference standards, and a WAZ < −3 SD respectively.

#### Skin erosion

A burn wound or another form of skin loss (e.g. skin loss as a consequence of severe napkin dermatitis) documented in the medical or nursing notes.

### Data analysis

Clinical data were extracted retrospectively from the hospital folders. Antibiotic sensitivity results were obtained from the NHLS microbiology database. The data were analysed using STATA Statistical software, release 11, (College Station, Texas, USA). Proportions were depicted as percentages. Continuous variables were tested for normality and mean and standard deviation (SD) or median and interquartile range (IQR) used to describe the data as appropriate. The Kruskal-Wallis equality of populations’ rank test or Wilcoxon rank-sum tests for independent samples were used to compare all continuous variables. Factors associated with the outcome risk of ESBL- infection or mortality were explored by univariate analysis. Generalised linear models using Poisson regression with robust error variance were used to estimate risk ratios and 95 % confidence intervals after controlling for potential confounding factors. A two sided significance level of *p* < 0.05 was used in all calculations. Infection risk was calculated per 1000 hospital admissions.

## Results

### Risk of *Klebsiella pneumoniae* bloodstream infections

**Fig. 1:**
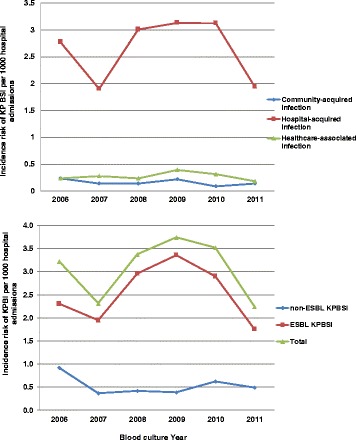
Incidence risk of Klebsiella pneumoniae bloodstream infection (KPBSI) per 1000 hospital admissions at RCWMCH January 2006- December 2011. Legend: ESBL-KPBSI: extended spectrum β-lactamase producing *Klebsiella pneumoniae* bloodstream infection

Over the 6-year study period, 410 hospitalised children had laboratory confirmed KPBSI, of whom 339 (83 %) had ESBL-KPBSI. The infection was hospital-acquired in 353, (86.1 %), healthcare-associated in 36, (8.8 %) or community-acquired in 21, (5 %). The annual risk of HA-KPBSI and ESBL-KPBSI increased from 2007, peaking in 2009 and then declining in 2011 (Fig. 1). Children hospitalised with CA-KP BSI were younger, compared to those with HA- or HCA-KPBSI, median age of 1.5 (IQR 0.7–3.8) months versus 5.5 (IQR 1.9–16.2) months, *p*-value = 0.01.

### Characteristics of the study population

The median age was 5.0 months (IQR 2 to 16 months), 198 were male and 212 female (Table 1). Two hundred and forty one (58.8 %) children were moderately or severely underweight-for-age (UWFA).

**Table 1:** Characteristics of the children with *Klebsiella pneumoniae* bloodstream infection

Variable	All	ESBL-KP^b^	Non-ESBL-KP	*p* value
Number of children (%)	*N* = 410	*n* = 339/410 (83 %)	*n* = 71/410 (17 %)	-
Male: female	212: 198	170: 169	1.77	0.17
Median (IQR) age in months	5 (2–16)	5 (2–5)	10 (2–29)	0.12
Median weight-for-age z-score (IQR)	−2 (−4 to −1)	−2 (−4 to −1)	−1 (−3 to −1)	0.02
Moderate under-weight-for-age n/N (%)	78/410 (19.0)	66/339 (19.5)	12 /71 (16.9)	0.14
Severe underweight-for-age n/N (%)	163/410 (39.8)	143/339 (42.2)	20 /71 (28.2)	0.01
HIV-infection^a^ (%)	(*n* = 288)	(*n* = 249)	(*n* = 39)	
	82 (28.5)	79 (31.7 %)	3 (7.7 %)	0.004
Exposure to a selection of antibiotics^c^	319/388 (82.2)	287/319 (90)	31/68 (46.4)	<0.0001
2nd - 4th generation Cephalosporins	205/388 (52.8)	190/205 (92.8)	15 /69 (17.8)	<0.0001
Aminoglycosides	227/388 (58.5)	206/319 (64.6)	21/69 (30.4)	<0.0001
Cotrimoxazole	96/388 (24.7)	91/319 (28.5)	5/69 (7.3)	0.001
Carbapenems	69/388 (17.8)	65/319 (20.4)	4 /69 (5.8)	0.004
Piperacillin-tazobactam	107/388 (27.6)	95/319 (29.8)	12/69 (17.4)	0.04
Fluoroquinolones	55/388 (14.2)	48/319 (15.1)	7/69 (10.1)	0.29

*KP Klebsiella pneumoniae*, *IQR* interquartile range, *n/N* stratum specific proportions

^a^HIV prevalence known in 288 children

^b^ESBL-KP: extended-spectrum-β-lactamase-producing *Klebsiella pneumoniae*

^c^Exposure to the following antibiotics within the last 12 months before the KP bloodstream infection: 2nd and/or 3rd and/or 4th generation cephalosporins, macrolides, fluoroquinolones, cotrimoxazole, aminoglycosides, piperacillin-tazobactam, and carbapenems

**Fig. 2:**
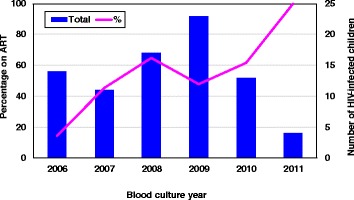
The number and percentage of HIV-infected children per annum with KP BSI on ART during the study period, 2006–2011. Legend: KP BSI-*Klebsiella pneumoniae* bloodstream infection; ART-Antiretroviral therapy

Two hundred and six (50.2 %) children were HIV negative, 82 (20 %) were HIV-infected, and the HIV-status was unknown in 122 (29.8 %) children. There were more HIV-infected children with moderate or severe UWFA compared to uninfected children, 61/82 (74.4 %), versus 126/206 (61.2 %), *p* = 0.03. The CD4-lymphocyte counts within the 3 month period preceding KPBSI were known in 71/82 (86.6 %) of all HIV-infected children. The median absolute CD4 count was 639 cells/mm^3^ (IQR 285 to 1235) and the mean CD4 percentage was 21.8 % (SD ± 12.33). Forty-two of 71 (59.2 %) HIV-infected children were severely immunosuppressed by CD4 percentage-for-age and 55/71 (77.5 %) severely immunosuppressed by absolute CD4 count-for age. Forty-one (50 %) of the eighty-two HIV-infected children were on antiretroviral therapy (ART) at the time of the KPBSI. The median time on ART before the KPBSI was 19 (IQR 8–66) days. The annual proportion of children on ART increased progressively over the study period (Fig. 2).

During the 12 month period preceding KPBSI, 40 % (155/387) of the children had received antibiotics such as 2nd – 4th generation cephalosporins, fluoroquinolones, macrolides, cotrimoxazole, aminoglycosides, piperacillin-tazobactam, and/or carbapenems. Those who acquired ESBL-KPBSI had higher preceding antibiotic exposure (Table 1). The median time from hospitalisation to KPBSI was significantly shorter for children with non-ESBL-KPBSI compared to those who developed ESBL-KPBSI, 3 (IQR 0–7) days versus 9 (IQR 4–23) days, *p* < 0.0001.

There were 29 (6.3 %) children who had either confirmed malignancy (26/29) or aplastic anaemia (3/29). Their mean age was 69.6 months (SD ± 50.29) and 41.4 %(12/29) were male. The infection was HA-KPBSI in 26/29 (90 %) children, caused by ESBL-producing KP in all but 3 children and occurred in the neutropenic phase in 20/29 (70 %) children. At the time of the KP-BSI, their median white cell count was 0.6 × 10^9^ /L (IQR 0.3–1.4).

Throughout the study period, HA-KPBSI caused by ESBL-producing isolates predominated (Table 2). Among children with CA-KPBSI, the number of ESBL-producing isolates remained low. Furthermore, there was a 47 % reduction in ESBL-KPBSI events between 2010 and 2011, from 63 to 37.

**Table 2:** Number and percentage of non-ESBL producing and ESBL producing *Klebsiella pneumoniae* BSI within each infection category 2006–2011

	Community-Acquired Infection		Hospital-acquired infection		Healthcare-associated Infection		Total
Year	ESBL KPBSI n (%)	Non-ESBL KPBSI n (%)	ESBL KPBSI n (%)	Non-ESBL KPBSI n (%)	ESBL KPBSIN (%)	Non-ESBL KPBSI n (%)	Total KPBSI n (100 %)
2006	3 (4.3)	2 (2.9)	46 (65.7)	14 (20.0)	1 (1.4)	4 (5.7)	70 (17)
2007	0	3 (6.0)	38 (76.0)	3 (6.0)	4 (8.0)	2 (4.0)	50 (12)
2008	3 (4.1)	0	59 (80.8)	6 (8.2)	2 (2.7)	3 (4.1)	73 (18)
2009	1 (1.2)	4 (4.7)	68 (79.1)	4 (4.7)	8 (9.3)	1 (1.2)	86 (21)
2010	0	2 (2.5)	63 (78.8)	8 (10.0)	3 (3.8)	4 (5.0)	80 (20)
2011	0	3 (5.9)	37 (72.6)	7 (13.7)	3 (5.9)	1 (2.0)	51 (12)
Totals	7 (1.7)	14 (3.4)	311 (76.0)	42 (10.0)	21 (5.1)	15 (3.7)	410 (100)

*ESBL KPBSI* extended-spectrum-β-lactamase-producing *Klebsiella pneumoniae* bloodstream infection, *BSI* bloodstream infection

### Spectrum of KP bloodstream infection

At the time of the KPBSI 70 % of children (284/405) experienced fever i.e., temperature of ≥38.0 °C. Although there was a wide range of clinical manifestations at the time of KPBSI, 80 % of the children either presented without a definable clinical focus or with pneumonia (Table 3).

**Table 3:** The clinical site of infection in children with *Klebsiella pneumoniae* bloodstream infection

Clinical site of infection	All	ESBL	Non-ESBL	*p* value
	n/N (%)	n/N (%)	n/N (%)	
No. with primary bloodstream infection without a definable focus	206/410 (50.5)	159/339 (46.9)	47/71 (66.2)	0.004
Common sites of infection				
Pneumonia	122 (29.8)	108 (31.9)	14 (19.7)	0.04
Vascular-catheter related	20 (4.9)	19 (5.6)	1 (1.4)	0.13
Peritonitis	20 (4.9)	16 (4.7)	4 (5.6)	0.76
UTI^a^	20 (4.9)	16 (4.7)	4 (5.6)	0.76
Septic wound and soft tissue	23 (5.7)	21 (6.2)	2 (2.8)	0.26
NEC^b^	20 (4.9)	18 (5.3)	2 (2.8)	0.37
Septic drip site (+ve^c^ culture of KP)	12 (2.9)	11 (3.2)	1 (1.4)	0.4
Cholangitis	4 (1.0)	4 (1.2)	0	N/S
Dysentery	3 (0.7)	2 (0.6)	1 (1.4)	0.4
Typhilitis	3 (0.7)	3 (0.9)	0	N/S
Bacterial endocarditis	1 (0.2)	1 (0.3)	0	N/S
Necrotic tumour	1 (0.2)	1 (0.3)	0	N/S
Bowel obstruction	1 (0.2)	1 (0.3)	0	N/S

*n/N* stratum specific proportions, *UTI* ^a^ urinary tract infection, *NEC* ^b^, necrotising enterocolitis, *+ve* ^c^ positive, *ESBL* extended-spectrum-β-lactamase-producing *Klebsiella pneumoniae*

### Risk factors for ESBL-associated KP infection

More children with ESBL-KPBSI were moderately or severely underweight than those who had non-ESBL KPBSI, i.e., 61.7 % (209/339) versus 45.1 % (32/71); *p* = 0.01; RR 1.13 (95%CI 1.02–1.24). Univariate analysis showed that infancy, concomitant HIV infection, cephalosporin exposure during the preceding 12 month period, mechanical ventilation prior to BSI, intravenous infusion for more than 3 days before the onset of KPBSI, an indwelling urinary catheter prior to KPBSI, major surgical procedure preceding KPBSI and a KP infection in the 12 months preceding the current KPBSI were significant risk factors for ESBL-KPBSI. On multivariate analysis HIV infection, cephalosporin exposure in the 12 month period before the KPBSI and continuous intravenous infusion for more than 3 days before the KPBSI remained significant risk factors for the acquisition of ESBL-KPBSI (Table 4).

**Table 4:** Factors associated with ESBL-*Klebsiella pneumoniae* bloodstream infection (BSI)

Risk factors for ESBL KPBSI	ESBL	Non-ESBL	Univariate analysis		Adjusted analysis (*n* = 271)	
	n/N (%)	n/N (%)	RR^a^ (95 % CI)	*p*-value	RR (95 % CI)	*p*-value
Moderate or severe underweight for age	209/339 (61.7)	32/71 (45.1)	1.13 (1.02–1.24)	0.01	0.99 (0.97–1.01)	0.40
HIV-infection	79/249 (31.7)	3/39 (7.7)	1.22 (1.13–1.30)	<0.001	1.14 (1.04–1.25)	**0.01**
Patient in the PICU^b^	92/339 (27.1)	11/71 (15.5)	1.11 (1.02–1.21)	0.04	0.99 (0.88–1.12)	0.94
Cephalosporin exposure in the last 12 months pre-BSI	184/339 (54.3)	12/71 (16.9)	1.30 (1.18–1.42)	<0.001	1.18 (1.06–1.31)	**0.002**
Mechanical ventilation prior to BSI	142/335 (42.4)	13/71 (18.3)	1.19 (1.10–1.29)	<0.001	1.08 (0.93–1.24)	0.31
Continuous infusion for >3 days prior to BSI	247/334 (74.0)	30/71 (42.3)	1.31 (1.16–1.49)	<0.001	1.15 (1.04–1.28)	**0.009**
Indwelling urinary catheter prior to BSI	139/335 (41.5)	12/71 (16.9)	1.20 (1.10–1.30)	<0.001	1.09 (0.99–1.20)	0.10
Major surgical procedure prior to BSI^c^	115/336 (34.2)	16/71 (22.5)	1.10 (1.01–1.20)	0.04	1.06 (0.95–1.18)	0.33
Skin erosions	111/336 (33.0)	19/71 (26.8)	1.05 (0.96–1.15)	0.30	1.07 (0.98–1.17)	0.12
Previous KP infection within last 12 months	39/338 (11.5)	3/71 (4.2)	1.14 (1.03–1.26)	0.01	0.96 (0.84–1.11)	0.62

Multivariable model adjusted for gender, age, HIV infection, underweight-for-age, mechanical ventilation, patient in the PICU at the time of the BSI, continuous IV infusion for more than 3 days before the BSI, indwelling urinary catheter, major surgery before the BSI, Previous KP infection within last 12 months, skin erosions and cephalosporin exposure during last 12 months before the BSI. aRR adjusted risk ratio

*ESBL-KP* extended-spectrum-β-lactamase-producing *Klebsiella pneumoniae*, *BSI* bloodstream infection

^a^RR risk ratio; ^b^PICU: paediatric intensive care unit; ^c^ A major surgical procedure included a laparotomy (emergency or elective), a thoracotomy, a skin graft or a craniotomy; Risk ratio 95 % confidence intervals that do not cross the null value of 1 are shown in bold

### Antibiotic sensitivity profile of the isolates

All isolates, both ESBL- (*n* = 339) and non-ESBL-producing (*n* = 71) were resistant to ampicillin. Of all isolates tested, 21 % (86/409) were sensitive to gentamicin, 76.1 % (312/410) were sensitive to amikacin, 60.5 % (211/349) were sensitive to piperacillin-tazobactam, and 29.1 % (117/402] were sensitive to co-amoxyclav. All isolates were sensitive to the carbapenems imipenem (409/409 tested) and meropenem (409/409 tested), and 99.5 % [403/405) were sensitive to ertapenem. The disaggregated antibiotic sensitivity patterns of the KP isolates are shown in Table 5.

**Table 5:** Antibiotic susceptibility results of extended spectrum beta lactamase (ESBL) and non-ESBL *Klebsiella pneumoniae* isolates

Antibiotic	Number of resistant isolates			
	Non-ESBL KP		ESBL KP	
	n/N	(%)	n/N	(%)
Ampicillin	71/71	(100)	339/339	(100)
Co-amoxyclav	5/71	(7.1)	280/331	(84.6)
Cefotaxime	0/71	(0)	339/339	(100)
Ceftazidime	0/71	(0)	339/339	(100)
Cefepime	0/71	(0)	339/339	(100)
Ciprofloxacin	0/71	(0)	142/339	(41.9)
Cotrimoxazole	18/71	(25.4)	313/339	(92.3)
Amikacin	0/71	(0)	98/339	(28.9)
Gentamicin	4/71	(5.6)	319/338	(94.4)
Piperacillin-Tazobactam	2/67	(3.0)	136/282	(48.2)
Ertapenem	0/67	(0)	2/338	(0.6)
Imipenem	0/71	(0)	0/338	(0)
Meropenem	0/71	(0)	0/338	(0)

*KP Klebsiella pneumoniae*, *ESBL KPBSI* Extended-spectrum-beta-lactamase-producing *Klebsiella pneumoniae* bloodstream infection, *co-amoxyclav* amoxicillin-clavulanic acid co-formulation, *n/N* stratum specific proportions

Of the 82 HIV-infected children, 38 (46.3 %) were on cotrimoxazole prophylaxis prior to the KPBSI and 96.3 % of their isolates (79/82) were ESBL-producing organisms. All (38/38) isolates of HIV-infected children on cotrimoxazole prophylaxis were resistant to cotrimoxazole as well as 37/39 (97 %) isolates of those who were not on cotrimoxazole prophylaxis. In 29 children with malignancy or aplastic anaemia, 20/29 (70 %) isolates were sensitive to piperacillin and/or amikacin.

### Antibiotic therapy

Data on antibiotic therapy were available for 398 children. Antibiotic therapy was considered appropriate if the laboratory reported the organism as susceptible to the specific antibiotic and suboptimal if the organism was reported as being intermediately susceptible or resistant. Empiric antibiotic therapy was appropriate in 307/398 (77.1 %) children comprising 241/328 (73.5 %) children with ESBL KPBSI and 66/70 (94.3 %) children with non-ESBL KPBSI. Piperacillin plus amikacin was the empiric antibiotic choice in 18/29 (62 %) children with malignancy or aplastic anaemia whilst 7/29 (24 %) received a carbapenem; 25/29 (86 %) children received a carbapenem as the definitive antibiotic. Once antibiotic susceptibility results became available, antibiotic therapy was adjusted in patients in whom empiric therapy was suboptimal. This delay did not alter the 30 day mortality significantly i.e. 26.4 % (74/280) of children on appropriate empiric therapy died versus 35.3 % (30/85) on delayed optimal therapy, *p* = 0.11, RR 0.75 (95 % CI: 0.53–1.06).

### KPBSI mortality

One hundred and twenty-three of the 410 study children died over the 6 year period, giving a crude case fatality rate of 30 %. Only the children dying within 30 days of the KPBSI (109/410, 26.6 %) were included in the mortality analysis as there was higher chance of identifying factors associated with KPBSI mortality in these children. Deaths occurring beyond this time period were deemed unlikely to be related to the KPBSI. The mean time to death from KPBSI diagnosis of the 14 children who died after 30 days was 46.4 (SD ±11.2) days. Among the 109 patients dying within 30 days, the median time to death (*n* = 109) was 3 days (IQR 1–9 days) (Table 6). One hundred (91.7 %) of the deaths were associated with ESBL-KPBSI. In 37.6 % (41/109) children the deaths occurred in children with ESBL KPBSI without a definable focus and 41.3 % (45/109) occurred in children who manifested with pneumonia. The KPBSI was community acquired in four (3.7 %) children who died, two of whom had non-ESBL KP isolates. Hospital-acquired (92/109, 84.4 %) and healthcare-associated (13/109, 11.9 %) KPBSI accounted for the rest of the mortality. Of the children who died 70.6 % (77/109) had a chronic underlying medical disorder, most commonly HIV infection (37 children), congenital cardiac conditions (11 children) and gastrointestinal conditions (14 children).

**Table 6:** Time to death in 3 time categories in study children with *Klebsiella pneumoniae* bloodstream infection

Cause of death	Time to death from KPBSI							
	≤3 days		3.1–14 days		14.1–30 days		Totals	
	n	(%)	n	(%)	n	(%)	n	(%)
Septicaemia	48	(44.0)	28	(25.7)	8	(7.3)	84	(77.1)
Pneumonia	6	(5.5)	4	(3.7)	5	(4.6)	15	(13.8)
Other	3	(2.8)	5	(4.6)	2	(1.8)	10	(9.1)
Totals	57	(52.3)	37	(33.9)	15	(13.8)	109	(100)

Factors contributing to 30-day mortality on univariate analysis included ESBL KPBSI, HIV infection, KPBSI requiring admission to PICU, and the presence of skin erosions, while KPBSI without a source had a protective effect. On multivariate analysis HIV infection, being in the PICU at the time of the BSI, KPBSI requiring admission to PICU, and the presence of skin erosions were significant risk factors for death (Table 7).

**Table 7:** Factors associated with 30-day inpatient mortality in children with *Klebsiella pneumoniae* bloodstream infection

Risk factors for death	Alive	Died	Univariate analysis		Adjusted RR^a^ for mortality (*n* = 259)	
	n/N (%)	n/N (%)	RR^a^ (95 % CI)	*p*-value	aRR (95 % CI)	*p*-value
ESBL-KPBSI	210/267 (78.7)	100/109 (91.7)	2.36 (1.26–4.43)	0.00	1.09 (0.55–2.16)	0.80
Moderate or severe underweight-for-age	150/267 (56.2)	71/109 (65.1)	1.31 (0.94–1.83)	0.11	1.12 (0.79–1.62)	0.54
HIV-infection**	38/181 (21.0)	37/83 (44.6)	2.03 (1.44–2.85)	<0.001	2.44 (1.59–3.74)	<0.001
Patient in the PICU at time of BSI**	56/267 (21.7)	31/109 (28.4)	1.28 (0.91–1.80)	0.16	1.64 (1.03–2.61)	0.04
Patient needing to go to PICU at the time of KP bloodstream infection**	44/267 (16.5)	34/109 (31.2)	1.73 (1.26–2.38)	0.001	1.72 (1.10–2.70)	0.02
Continuous IV infusion for >3 days before the BSI	174/264 (65.9)	73/108 (67.6)	1.06 (0.75–1.48)	0.76	0.93 (0.64–1.35)	0.70
BSI without source**	145/266 (54.5)	41/108 (38.0)	0.61 (0.44–0.86)	0.004	0.82 (0.55–1.22)	0.33
Chronic underlying medical condition excluding HIV infection	124/267 (46.4)	41/109 (37.6)	0.77 (0.56–1.07)	0.12	1.30 (0.81–2.08)	0.28
Skin erosions**	70/265 (26.4)	47/108 (43.5)	1.69 (1.24–2.30)	0.001	2.15 (1.54–3.0)	<0.001

*n/N* stratum specific proportions; **p-value ≤ 0.05; *RR* risk ratio

*ESBL KP BSI* Extended-spectrum-beta-lactamase-producing *Klebsiella pneumoniae* bloodstream infection, *PICU* paediatric intensive care unit, *aRR* adjusted risk ratio *:Multivariate model adjusted for age, gender, nutrition, HIV infection, ESBL, Patient in PICU, patient needing to go to PICU, continuous IV infusion for >3 days before the BSI, KPBSI without source, chronic underlying medical condition excluding HIV, and skin erosions

## Discussion

In this hospital based retrospective study we have described laboratory-confirmed *Klebsiella pneumoniae* bloodstream infections in children admitted to RCWMCH from 1 January 2006–31 December 2011. This is the first comprehensive analysis of *Klebsiella pneumoniae* bloodstream infections in hospitalised children at a dedicated children’s hospital in Sub-Saharan Africa. This study showed that ESBL- *Klebsiella pneumoniae* is an important cause of HA- and HCA-BSI and is associated with high mortality.

Notably 68.9 % of the children were less than 12 months old. In the only other large published paediatric case series on *KPBSI* that involved 57 American children, infancy was also the predominant age-category i.e., 68 % (*n* = 38/57) of the children were less than 12 months [10]. Young children, particularly infants, are not fully immunocompetent and more susceptible to bacterial infections including KPBSI. Young age and immunological immaturity may also contribute to the delayed diagnosis of sepsis [16]. A high prevalence of moderate and severe underweight, an indicator of malnutrition was documented. On univariate analysis moderate or severe underweight-for-age was more frequent in children with ESBL-KPBSI (209/339, 61.7 %) compared to those with non-ESBL KPBSI (32/71, 45.1 %); *p* = 0.01. Malnutrition is known to be associated with widespread micronutrient deficiencies, immune dysfunction, and susceptibility to infectious complications [17].

The study population was representative of the children living in the Western Cape region, coming mainly from peri-urban, low socio-economic sub-districts. National figures show that the incidence of under-weight-for-age (UWFA) in children <5 varied from 16/1000 (2012) to 22/1000 (2009) and that the incidence of severe acute malnutrition was 4-5/1000 (2009–2012) [18]. The 2011 national Census established that 40 % of 5 million South African children <5 years-of-age were UWFA and that there was a high incidence of stunting (26.9 %) in children <3 years-of age [19, 20]. Concomitant HIV infection is known to be a significant contributor to malnutrition and was present in 20 % of the study children.

At the time of the positive blood culture 30 % (126/410) of the study children did not experience fever and 50.2 % had no definable clinical focus. When a focus was present this was commonly in the lungs. In a parallel study on *Staphylococcus aureus* bloodstream infection at our hospital, 33 % of children had no identifiable source and, where one was identified, the lungs were the most common focus (22 %) [21]. In another paediatric study describing BSIs caused by of Gram-negative pathogens including *KP*, the absence of a clear clinical focus occurred in 68 % of the study subjects [6]. Unlike a child presenting from home with a CA-BSI where the clinical manifestations precipitate presentation to a health facility, HA-BSI may manifest with non-specific signs leading to delay in recognising active infection. Furthermore, the decision to take blood cultures were made by the caring clinicians based on clinical indications; given the retrospective study design no attempt was made to interrogate the possibility of blood culture contamination.

In the current study KPBSI was primarily a hospital-acquired infection caused by ESBL-producing isolates. Community-acquired infection was uncommon, particularly community-acquired infection caused by ESBL-producing isolates. These findings are similar to previous studies describing Gram-negative BSI in children [6, 22]. Prior cephalosporin exposure increased the risk for ESBL-KPBSI, confirming findings of previous paediatric studies [2–5]. Two additional statistically significant risk factors were identified. The risk of HIV infection probably relates to the underlying immunodeficiency, particularly as 50 % of the HIV-infected children were not on ART at the time of acquiring KPBSI. The risk of continuous intravenous infusion for more than 3 days before the BSI was diagnosed may result from poor infection control practice and / or contamination of the infused fluid during intravenous drug administration. Published studies have implicated these factors, but no paediatric study has previously shown a statistical relation between BSI and prior intravenous infusion on multivariate analysis [23, 24].

At the time of the study, empiric antibiotic therapy at RCWMCH for CA-BSI was ampicillin plus gentamicin and for HA- and HCA-BSI, piperacillin-tazobactam plus amikacin. These empiric regimens were also applied to children who had malignancy or aplastic anaemia. The study findings suggest that these empiric antibiotic combinations would not have been effective against 22.9 % of non-susceptible isolates, intermediate or resistant, in patients with KPBSI. Once antibiotic susceptibility results were made available to the attending clinicians these patients were started on effective antibiotic therapy, and the resultant delay did not significantly affect 30-day mortality. However, the low cover for HA- and HCA-BSI suggest that more effective empiric antibiotic therapy using a carbapenem should be considered. This suggestion concurs with previous studies [3, 5]. During the study period there was full access to all classes of appropriate antibiotics that were reported by the NHLS microbiology laboratory including ertapenem, imipenem and meropenem.

There was a notable surge and decrease in the risk of ESBL-producing KP HA-infection over 2009 and 2010. The impact of a South African measles epidemic starting in the Western Cape Province in 2009, peaking during a diarrhoeal surge season in March and April of 2010 and then abating in November of the same year was associated with severe pressure on beds and subsequent overcrowding in all wards of the hospital, this may have accounted for the surge seen. The falling trend in the risk thereafter at the hospital may well also reflect some success of the preventive strategy package driven by the hospital infection control unit e.g., increased awareness of the importance of hand hygiene, training on how to perform hand hygiene and an effort to reduce overcrowding in certain key areas of the hospital.

The impact of Gram-negative bacteraemia is significant in children with reduced immunity, including those with cancer, and access to carbapenem antibiotics is important particularly as the control of sepsis during febrile neutropenic episodes is time-critical.

The high 30-day fatality rate of 26.6 % and with 52.3 % of the deaths occurring within 3 days of the positive blood culture result were consistent with previous paediatric studies of Gram-negative BSI including *KP* where mortality ranged from 13 to 36 % [2, 3, 8–10] and a bacteraemia study in rural Kenya in which 70.5 % of deaths occurred within 3 days of positive blood culture [25]. In the present study HIV infection, being in the PICU at the time of BSI or needing PICU admission and the presence of skin erosions were significant risk factors for death. Previous paediatric studies have identified a different spectrum of independent risk factors for death including the presence of an ESBL-producing organism, shock on admission to hospital and more than 48-h delay in initiating appropriate antibiotic therapy, and in neonates infectious complications after the onset of BSI and pulmonary hypertension. In the present study, mortality was higher in children who received suboptimal empiric antibiotic therapy (35.3 % vs 26.4 %). Although this difference appeared clinically relevant, it did not achieve statistical significance, partly because the analysis was underpowered on post-hoc evaluation i.e., power of 36 % at an alpha of 0.05.

### Study strengths and limitations

A strength of this study is that it is the first large African study to describe KPBSI in children, providing clinical and epidemiological information which was not previously available. Because of the retrospective study design, there were limitations in the availability and completeness of clinical and laboratory data. For example, the HIV status of 122 children was not known because there was no documentation of testing in the clinical notes or at the laboratory. Were these results available they would have influenced the risk factor analyses. One further strength of the study was that the ESBL status of the KP isolates did not influence the extraction of data from the folder review because the study team were blinded to status at the time of the data collection. The true burden of community-acquired KPBSI, particularly non-ESBL KPBSI events may have been underestimated. Before sick children are referred from primary or secondary health facilities to RCWMCH, they are often administered parenteral broad-spectrum antibiotics consistent with the World Health Organization Integrated Management of Childhood Illness guidelines that have been adapted for use in South Africa [26]. This practice frequently results in negative blood culture results. Further, due to the retrospective design the history and detail of exposure to previous hospitalisation and antibiotics was limited to what was documented in the case folders by the admitting doctors and any pharmacy prescription sheets that were available; any outpatient exposures and admissions to hospitals other than RCWMCH that were not recorded in the folders were not captured and could have led to potential under reporting as exposed to antibiotics. Because of the limitations associated with laboratory reporting, for the purposes of this study, *KP* was assumed to be an ESBL producer if it was reported to clinicians as resistant to all cephalosporins. The laboratory continued to report ESBL- producing Enterobacteriaceae as resistant to all cephalosporins, despite changes in reporting practices introduced by CLSI in 2010, hence there may have been over reporting of cephalosporin resistance in the latter years. Due to limitations in laboratory interfaces MIC data are not routinely available to clinicians.

Although the results of this study may not be generalizable to other referral hospitals or community facilities, they demonstrate that KPBSI is an important childhood infection and substantially improved infection control practice is required to reduce the burden of hospital-acquired KPBSI.

## Conclusion

Multi-drug resistant *Klebsiella pneumoniae* bloodstream infection is associated with high mortality in children. Our analysis together with previous studies suggest that this infection is strongly associated with hospitalisation. Prospective studies are required to further explore the clinical and epidemiological observations documented in this study, and characterise the microbiological features and relatedness of KP isolates causing BSI in our setting.

## Abbreviations

aRR: Adjusted risk ratio
ART: Antiretroviral therapy
BSI: Bloodstream infection
CA: Community-acquired
CLSI: Clinical Laboratory Standards Institute
CVP: Central venous pressure
ESBL KP: Presumed extended spectrum β-lactamase producing *Klebsiella pneumoniae*
ESBL: Extended spectrum β-lactamase
HA: Hospital-acquired
HCA: Healthcare-associated infection
HIV: Human immunodeficiency virus
IQR: Interquartile range
KP: *Klebsiella pneumoniae*
*KPBSI*: *Klebsiella pneumoniae* bloodstream infection
LIS: Laboratory information system
NEC: Necrotising enterocolitis
NHLS: National Health Laboratory Service
PICU: Paediatric intensive care unit
RCWMCH: Red Cross War Memorial Children’s Hospital
SD: Standard deviation
UTI: Urinary tract infection
UWFA: Underweight-for-age
WAZ: Weight-for-age z-score
WHO: World Health Organisation
